# Adjunctive low‐level laser therapy in periodontal treatment – A randomized clinical split-mouth trial

**DOI:** 10.1007/s00784-025-06289-2

**Published:** 2025-04-25

**Authors:** Selma Dervisbegovic, Stefan Lettner, Dino Tur, Markus Laky, Apostolos Georgopoulos, Andreas Moritz, Anton Sculean, Xiaohui Rausch-Fan

**Affiliations:** 1 Clinical Division of Periodontology, University Clinic of Dentistry, Medical University of Vienna, Vienna, Austria; 2 Karl Donath Laboratory, University Clinic of Dentistry, Medical University of Vienna, StatisticsVienna, Austria; 3 Core Facility Oral Microbiology and Hygiene, University Clinic of Dentistry, Medical University of Vienna, Vienna, Austria; 4 Department of Periodontology, School of Dental Medicine, University of Bern, Bern, Switzerland; 5 Center for Clinical Research, University Clinic of Dentistry, Medical University of Vienna, Vienna, Austria

**Keywords:** Low-level laser, Periodontitis, Periodontal disease, Randomized controlled trial, Phototherapy

## Abstract

**Background:**

Low‐level laser therapy (LLLT) has been shown to exert biostimulatory effects, including increased cell proliferation and accelerated wound healing. Hence, the use of LLLT as an adjunct to scaling and root planing (SRP) for improved periodontal treatment outcomes has been examined. The aim of this study was to evaluate the clinical effect of adjunctive LLLT in the treatment of periodontitis with a 980-nm diode laser.

**Materials and methods:**

Patients with a periodontal screening index of 3 or 4 who met the inclusion criteria were recruited and randomized into two groups for treatment allocation in a split-mouth design. The maxillary and mandibular left or right quadrants of the patients were assigned to either the test (SRP + LLLT) or the control (SRP) group. During the two final debridement sessions, LLLT was applied on one side of each study participant´s upper and lower jaws. The reevaluation of the clinical parameters and microbiological assays was performed 12 weeks after the initial therapy.

**Results:**

Both groups presented significant reductions in clinical parameters (p < 0.001). However, no statistically significant differences between the test and control groups were found for any of the parameters (all p values were greater than 0.05). The recolonization of *P. gingivalis* and *T. denticola* was not significantly reduced in the laser group.

**Conclusion:**

The clinical parameters in both groups improved similarly after initial periodontal treatment. LLLT with the chosen settings did not show a beneficial effect during the initial nonsurgical treatment of periodontitis.

**Clinical relevance:**

LLLT is under discussion for periodontal therapy as a promising treatment modality. Compared with nonsurgical therapy alone, adjunctive periodontal treatment with a diode laser did not improve the clinical parameters of periodontitis patients in this study. Currently, there is no recommended treatment protocol for adjunctive LLLT in periodontitis, which needs to be further investigated with other laser settings.

**Clinical trial registration:**

ISRCTN registry (#ISRCTN11275257), retrospectively registered, 16.10.2023.

## Introduction

Low-level laser therapy (LLLT) is a noninvasive treatment modality that has been used for medical conditions since the 1960s [1]. The main applications of LLLT, which has been used for more than two decades, include musculoskeletal pain relief as well as the promotion of wound healing through the reduction of the inflammatory process, the modulation of growth factors and increased angiogenesis [1–3]. This treatment modality results in the modulation of growth factors and myogenic regulatory factors and effectively promotes sciatic nerve regeneration. The low-level lasers are absorbed by chromophores, which leads to a modulation of pathophysiological processes. Additionally, bacterial growth inhibition at low-level doses has been reported in vitro [4]. The devices suitable for LLLT are class 3B lasers with a mean output power ranging from 5 to 500 mW [2]. The lasers recommended for this treatment are those emitting in the red and near-infrared spectra, which are mainly diode lasers that induce photobiomodulary reactions [5]. The radiation is either emitted in a continuous wave or in pulsed mode with a low energy density of up to 50 J/cm2 [6]. The effects of LLLT are mostly achieved via nonthermal photobiological mechanisms. Laser light that is emitted in the range of the “optical window” (600–1100 nm) penetrates deeper into the tissue and induces a wider cell-light response [6, 7]. Arndt–Schultz’s curve describes the biphasic dose response and thus poses an important point to consider when creating a photobiomodulation protocol [8].

Periodontitis is an inflammatory disease characterized by severe attachment loss and is considered to be a major cause of tooth loss in adults. According to a recent oral health study, every second adult is affected by periodontitis over the age of 35, with up to 42% of patients suffering from moderate disease and every tenth patient suffering from a severe form of periodontal disease [9].

The response of the immune system to periodontal pathogens results in alveolar bone resorption via various mechanisms, which medical science has yet to fully comprehend. Periodontitis can be categorized according to the new classification into stages I to IV and grades A, B and C [10] according to the severity, extent and progression of the disease [11, 12]. Nonsurgical therapy for periodontitis involves biofilm disruption and removal from tooth surfaces, which is important for enabling the reattachment of adjacent periodontal tissues. Periodontal pathogens colonizing the periodontal pockets penetrate other cells outside the epithelial pocket, where they may remain after biofilm removal and recolonize the periodontal pocket [13]. Hence, bacteria can evade the immune response of the host by releasing virulence factors and thereby enhancing the inflammatory process. [14, 15]. Systemic antibiotics can be indicated in some severe cases and are prescribed as an adjunct to nonsurgical therapy. With rising levels of resistance among periodontal pathogens [16], it is recommended that this medication be administered sensibly and restrictedly as an adjunct to patients who are likely to gain a clinical benefit [17]. Alternative adjuvant treatment options are the subject of current research in the field of periodontology and could improve the outcome of nonsurgical therapy. Recently, laser therapy has been considered the new gold standard for periodontal pocket treatment [18]. LLLT has been applied in recent studies as an adjunct to initial periodontal treatment; however, the results have been inconsistent due to various experimental settings and parameters. This treatment modality was reported to be safe for the vitality of dental tissue since it does not result in increased tissue temperature [19, 20]. LLLT has been shown to improve clinical attachment levels as well as bleeding, plaque and gingival indices compared with scaling and root planing alone [21–24]. The objective of this randomized controlled trial was to further examine the adjuvant benefit of low-level laser therapy in patients with moderate to severe periodontitis utilizing a split-mouth research design with a 980-nm diode laser. The working hypothesis of this study was that LLLT could enhance clinical parameters in the initial phase of periodontal treatment.

## Material and methods

### Study population

The study protocol was approved by the Ethics Committee of the Medical University of Vienna (EK Nr.: 2241/2016). The study was performed in accordance with the Helsinki Declaration of 1975, which was revised in 2013. The study participants had to meet the following criteria with regard to the old classification of periodontitis [25]: localized or generalized moderate to severe periodontitis with a periodontal screening index of 3 or 4 [26]; with regard to the new classification of periodontitis [12], localized or generalized periodontitis was diagnosed with periodontitis stage II, III or IV with grades B or C. Additional inclusion criteria were as follows: aged 25–55 years with the presence of at least one site in each quadrant with probing depths above 5 mm with bleeding on probing, radiologically detectable alveolar bone loss in all quadrants and good general health. The patients were diagnosed through evaluation of panoramic X-ray images and were included in the trial if they presented with a minimum degree of bone loss of 15% and a CAL of 3 mm or more. Patients were considered to be in good general health when no severe systemic condition, apart from periodontitis, was present. The exclusion criteria were current pregnancy; the use of systemic or local antibiotic treatment in the last 6 months; active periodontal treatment during the last 6 months; the presence of an infectious disease; chronic pulmonary disease; cancer or diabetes; other apparent oral infections; and the intake of immunosuppressive medication or immunodeficiency.

The study participants were informed about the study protocol and possible side effects of the treatment and signed a written consent form.

### Calculation of sample size

The sample size was calculated on the basis of previous studies [27, 28]. A standard deviation of 15 percentage points for bleeding on probing between treatment groups was assumed for the split-mouth design. A difference of 10 percentage points for bleeding on probing was considered clinically relevant. We calculated that 20 patients were needed for the detection of a difference of 10 percentage points for bleeding on probing with 80% power at an alpha level of 0.05.

### Examiner calibration

Two calibrated examiners, S.D. and D.T., performed the clinical examination. The clinical parameters included PPD, CAL and BoP, which were measured with a calibrated standard probe (CP 12, Hu-Friedy, Chicago, IL, USA). For the calibration, clinical parameters were obtained at 42 randomly chosen sites, and the intraclass correlation coefficient (ICC) for the PPD was 0.916.

### Clinical examination and treatment

Patients were recruited at the University Clinic of Dentistry in Vienna from July 2017 to August 2022 by one of the examiners after the initial periodontal examination. Patients with a Periodontal Screening Index (PSI) of 3 or 4 who fulfilled the listed inclusion criteria were asked to participate in the study [26]. The patients were numbered according to the date of recruitment and were randomized into two groups according to a randomization list (https://www.randomizer.org) for treatment allocation in a split-mouth design. The maxillary and mandibular left or right quadrants of the patients were assigned to either the test group (SRP + LLLT) or the control group (SRP). Before treatment, the patient´s general medical and dental history was recorded, and a panoramic X-ray image was obtained. The initial treatment consisted of periodontal charting and individual oral hygiene instructions. The patients received oral hygiene instructions with toothbrushes and adequate interdental cleaning devices. For assessment of the patient´s oral hygiene, the Approximal-Plaque-Index (API) (Lange 1986) [29] and the Papillary Bleeding Index (PBI) (Saxer and Muehlemann 1975) [30] were obtained. A complete periodontal chart was recorded by experienced, calibrated examiners. The measurement of the periodontal probing depth (PPD) and clinical attachment level (CAL) was performed with a calibrated standard probe to the nearest millimetre at six sites of each tooth: disto-buccal, mesio-buccal, mid-buccal, mid-palatinal/lingual, mesio-palatinal/lingual and disto-palatinal/lingual. Bleeding on probing (BOP), plaque and suppuration were recorded, if present.

After the periodontal assessment, the initial treatment was performed in 2–4 supra- and subgingival debridement sessions using sonic instruments (Sonicflex 2003 KaVo, Biberach, Germany), universal curettes and Gracey curettes (HU-Friedy Co., Chicago, IL, USA).

The patients subsequently received oral hygiene controls to optimize individual plaque control during the initial stage of periodontal treatment. Scaling and root planing were performed in both groups. During the two final debridement sessions, the laser was applied on one side of each study participant’s upper and lower jaws. The contralateral sides did not receive adjunctive laser treatment and served as the control group. The laser used in this study was the Denlase Diode Laser, which emits at 980 nm, and the treatment was performed in accordance with the manufacturer´s protocol. Sites with periodontal pockets were treated with the laser for 60 s at a distance of 0.5 mm from the gingiva, with a maximum energy density of 21.7 J/cm^2^. The test sites of the treatment were not revealed to the patient. To simulate LLLT at the control sites, the operator conducted a sham procedure with the inactive laser device in the nontreated areas. The laser treatment was performed by the same experienced examiner. All participants and the examiner were provided with protective eyewear during laser application.

The reevaluation of the clinical parameters was performed 12 weeks after the initial therapy by a second calibrated examiner, who was blinded to the patient´s treatment.

The study participants were assigned a sequential code number (pseudonymized). The data were analysed by a blinded examiner and stored on an Excel sheet on a PC with access limited to authorized personnel of the Clinical Division of Periodontology.

### Microbiologic examination

After the initial examination and at the reevaluation, samples of subgingival plaque from the four deepest pockets in every quadrant were collected with ISO 20 paper points for a duration of 10 s. Prior to sampling, the supragingival plaque was removed, and the sites were isolated with cotton rolls. The samples from the test side and the control side were pooled separately and transported in a carrier medium to the microbiology laboratory for evaluation. The analysis was performed in the Core Facility Oral Microbiology and Hygiene (University Clinic of Dentistry, Medical University of Vienna, Austria) within one week after the plaque samples were obtained. The samples were stored at −20 °C before analysis. A polymerase chain reaction (PCR) detection method with a semiquantitative, commercially available DNA probe test kit (micro-IDent Plus, Hain Lifescience, Nehren, Germany) was used to determine the prevalence of *A. actinomycetemcomitans, P. gingivalis, T. forsythia, T. denticola, P. intermedia, P. micros, F. nucleatum, C. rectus, E. nodatum, E. corrodens, and Capnocytophaga spp.* The detection limit for *A. actinomycetemcomitans* was 10^3^, whereas for the remaining bacteria, it was 10^4^. The method is based on a DNA strip technique with multiple rounds of PCR and subsequent reverse hybridization.

### Statistical analysis

The primary outcome of this trial was BoP, and the secondary outcomes were CAL, PPD, gingival recession and microbial counts. The API and PBI were obtained and calculated for both sides rather than for each group separately. The analysis was performed following a similar strategy to that used in the investigation of Husejnagic et al. [28], which had a comparable study design and similar endpoints. Boxplots for CAL and PPD and bar charts for the results of microbiological examinations are shown. For inference, we used linear mixed models [31], including fixed effects for the treatment groups as well as the baseline, sex, age, smoking status, API and PBI, and tested random effects of position within teeth within patients. The dependent variables in these models were CAL and PPD. For the dependent variable (BOP), a generalized logit version of the same model was used [31] and further extended to ordinal logit mixed models [32] for microbial measurements. All calculations were performed in R version 4.3.0 [33]. For graphics, we used the package ggplot2 [34]. P ≤ 0.05 was considered statistically significant.

### Examiner calibration

Two calibrated examiners, S.D. and D.T., performed the clinical examination. The clinical parameters included PPD, CAL and BoP, which were measured with a calibrated standard probe (CP 12, Hu-Friedy, Chicago, IL, USA). For the calibration, clinical parameters were obtained at 42 randomly chosen sites, and the intraclass correlation coefficient (ICC) for the PPD was 0.916.

## Results

Thirty patients were enrolled in the trial. Three participants did not receive laser treatment due to antibiotic therapy following recruitment, and their data were excluded. After therapy, an additional seven individuals were lost to follow-up. For this study, the results of a total of 20 patients were analysed, with 1692 sites in the test group and 1704 sites in the control group (Table 1).

**Table 1:** Number of patients, teeth and sites in the test (SRP + LLLT) and control (SRP) groups; sex, smoking status, age of the participants; Baseline, beginning of treatment; Reevaluation, 3 months after treatment; BoP, bleeding on probing; CAL, clinical attachment level; GR, gingival recession; LLLT, low-level laser therapy; PPD, periodontal probing depth; SD, standard deviation; SRP, scaling and root planing

	Treatment	Patients	Teeth	Sites
No #	SRP	20	320	1704
	SRP + LLLT	20	319	1692
		**male**	**female**	
Gender	SRP	11	9	
	SRP + LLLT	11	9	
		**yes**	**no**	
Smoker	SRP	7	13	
	SRP + LLLT	7	13	
		**Mean**	**SD**	
Age (Years)	SRP	43,9	8,8	
	SRP + LLLT	43,9	8,8	
BoP Baseline (%)	SRP	19,4	12,5	
	SRP + LLLT	20,1	10,6	
BoP reevaluation (%)	SRP	9,8	10,1	
	SRP + LLLT	9,6	10,1	
PPD Baseline (mm)	SRP	3,38	1,8	
	SRP + LLLT	3,54	1,86	
PPD reevaluation (mm)	SRP	2,91	1,36	
	SRP + LLLT	2,94	1,34	
GR Baseline (mm)	SRP	0,13	0,57	
	SRP + LLLT	0,12	0,54	
GR reevaluation (mm)	SRP	0,21	0,73	
	SRP + LLLT	0,20	0,71	
CAL baseline (mm)	SRP	3,53	1,86	
	SRP + LLLT	3,68	1,90	
CAL reevaluation (mm)	SRP	3,15	1,58	
	SRP + LLLT	3,18	1,54	

A flow diagram of the stages of this study is presented in Table 2. The mean age of the study participants was 43.90 ± 8.8 years, with 7 smokers and 13 nonsmokers participating in the trial. The total number of treated pockets is shown in Table 1.

**Table 2.**
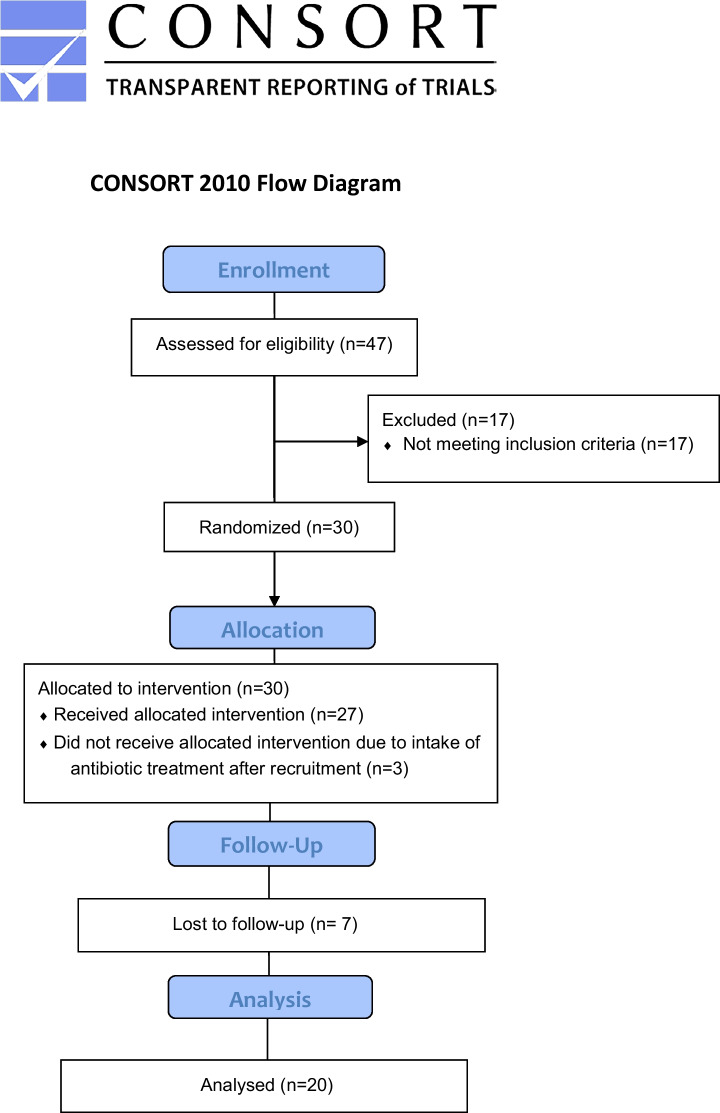
Flow chart of the progress through the phases of this randomized clinical trial of two groups (CONSORT 2010 Statement)

**Fig. 1:**
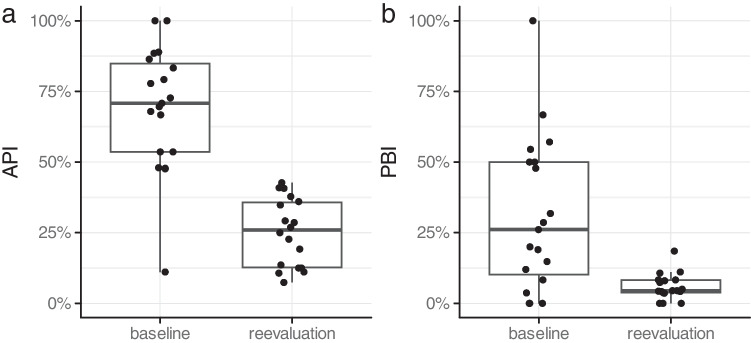
** a** Plaque values presented as percentages at baseline and reevaluation for both groups. API, Approximal Plaque Index; Baseline, beginning of treatment; Reevaluation, 3 months after treatment; SRP, scaling and root planning; **b** Percentages of papillary bleeding at baseline and reevaluation in both groups. Baseline, beginning of treatment; PBI, papillary bleeding index; reevaluation, 12 weeks after treatment; SRP, scaling and root planing

The clinical parameters obtained at baseline were similar for both groups, with no significant difference in smoking status. The API value was 69.13 ± 21.94 at baseline and improved to 25.13 ± 11.92 after treatment (Fig. 1A). The PBI score at baseline was 31.07 ± 27.22 and was reduced to 5.91 ± 4.57 at reevaluation after 3 months (Fig. 1B). The oral hygiene indices were not compared between the groups because of the split-mouth study design.

**Fig. 2:**
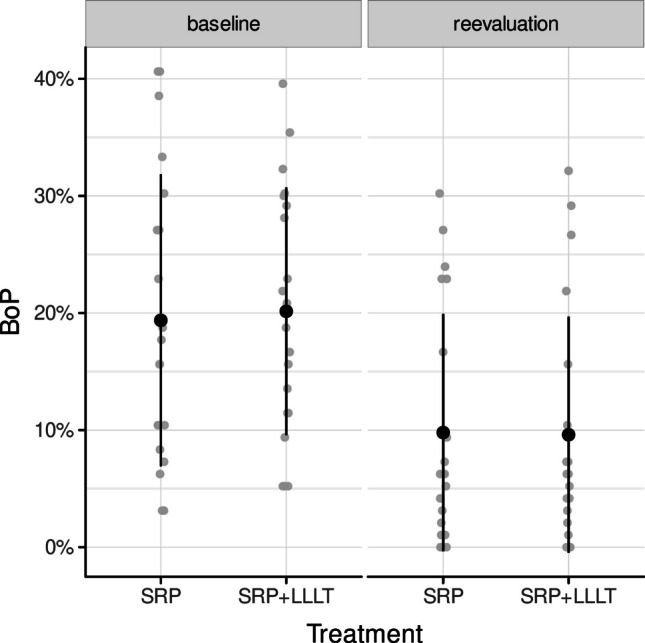
Distribution of bleeding on probing values as percentages for baseline and reevaluation in both treatment groups; baseline, beginning of treatment; BOP, bleeding on probing; LLLT, low-level laser therapy; reevaluation, 3 months after treatment; SRP, scaling and root planing

**Fig. 3:**
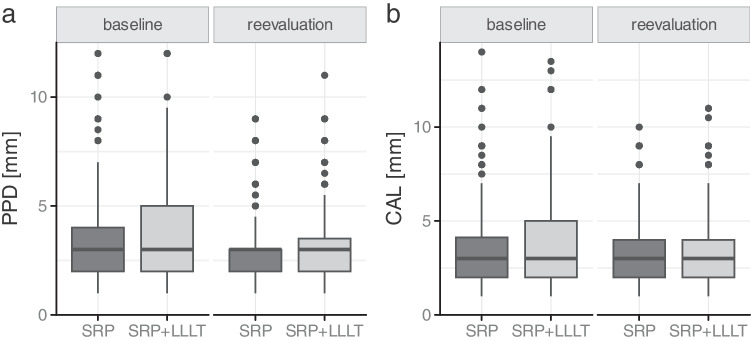
** a** Distribution of periodontal probing depth at baseline and reevaluation in both treatment groups; Baseline, beginning of treatment; LLLT, low-level laser therapy; PPD, periodontal probing depth; Reevaluation, 3 months after treatment; SRP, scaling and root planing; **b** Distribution of clinical attachment levels at baseline and at reevaluation in both treatment groups; baseline, beginning of treatment; CAL, clinical attachment level; LLLT, low-level laser therapy; reevaluation, 3 months after treatment; SRP, scaling and root planning

**Fig. 4:**
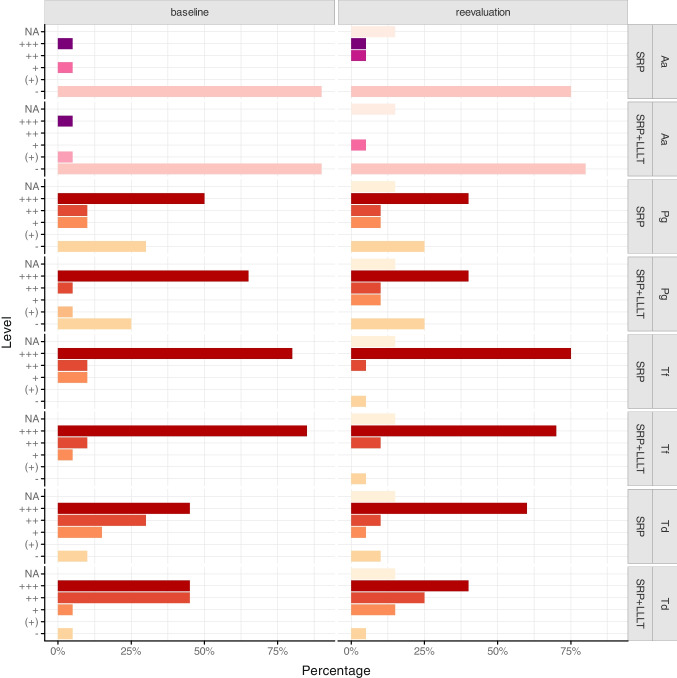
Detection levels of four major periopathogenic bacteria in periodontal pockets as percentages at baseline and reevaluation in both treatment groups. Baseline, beginning of treatment; Reevaluation, 3 months after treatment; Aa, *A. actinomycetemcomitans; *LLLT, low-level laser therapy; Pg, *P. gingivalis*;SRP, scaling and root planing; Tf,* T. forsythia*;Td,* T. denticola*

The clinical findings of the periodontal chart are presented in Figs. 2 and 3, with no significant differences between the groups. Therapy improved the BoP, PPD, and CAL in both groups. The microbiological findings predominantly indicated a decrease in periopathogenic microorganisms at reevaluation relative to baseline. We observed a somewhat reduced bacterial load of *P. gingivalis and T. denticola* following laser therapy; nevertheless, this difference was not statistically significant (Fig. 4). Throughout the experiment and subsequent treatments, no subjects reported side effects or adverse reactions.

## Discussion

Greater cell proliferation and wound healing are two reported biostimulatory effects of laser irradiation [1, 3], which may enhance clinical outcomes during periodontal therapy. Previous in vitro studies have suggested that red and infrared LLLT may exhibit dose-dependent anti-inflammatory effects [35]. This effect could be beneficial for the treatment of periodontitis to enhance the wound healing process and could be a possible treatment choice for immunocompromised patients or nonresponsive sites. LLLT has been shown to promote angiogenesis and control proliferation during the inflammatory process, thereby increasing the number of processes involved in tissue repair [36]. A recent in vitro study suggested that low-level lasers emitting at 660 nm and 780 nm can modulate the cellular activation status of macrophages in the context of inflammation [37]. The results of this study, however, did not reveal any differences between the LLLT group and the control group. Both groups showed improvement in the clinical parameters, with a reduction in probing depth and bleeding values and an increase in attachment. In terms of the bacterial load in the periodontal pocket, a greater reduction in *P. gingivalis* and *T. denticola* was detected in the laser group than in the control group, but the difference was not statistically significant. The primary aim of the current study was to analyse potential improvements in clinical parameters. However, previous studies included biochemical assays or gene expression analysis with a short-term reduction in inflammatory markers in the gingival crevicular fluid (40). LLLT can be utilized in both contact and noncontact modalities. An angled LLLT probe is advised for intraoral noncontact application of the device. A sufficient number of sites must be encompassed by the probe while adhering to the laser beam's radius to address the full area of inflammatory periodontal tissue. This method of treatment appears to be painless and highly acceptable to patients. In addition to the adequate device and indications, time is a prerequisite for choosing this adjunctive therapy, depending on the number of sites. Hence, repeated adjunctive phototherapy seems to have a beneficial effect on the reduction of inflammatory mediators over a single treatment [38]. For this study, LLLT was administered twice in noncontact mode.

Scaling and root planing (SRP) results in a reduction in the number of microorganisms; however, this treatment does not eliminate the pathogenic bacteria completely from the periodontal pocket [13]. A recent study investigated the effects of blue lasers on bacterial growth in vitro in the main species that usually colonize cutaneous ulcers. The authors observed an inhibition for *E. coli, P. aeruginosa* and *S. aureus* after LLLT at a maximum fluence of 24 J/cm^2^ [4]. The results of Petrovic et al. revealed a statistically significant decrease in the prevalence of bacteria in subgingival samples and in clinical parameters after treatment with adjuvant LLLT compared with SRP alone [39]. In the present study, the microbiological results revealed a greater reduction in *P. gingivalis* and *T. denticola* in the laser group than in the control group, which was not statistically significant, and a larger sample size is needed for further investigation. Despite conservative periodontal treatment, periopathogenic microorganisms infiltrate other cells outside the epithelial pocket and may persist there, avoiding the host's immune response and the effects of antimicrobial agents [14, 15], hence recolonizing the periodontal pockets. In this study, plaque samples were collected at baseline and three months posttreatment. When interpreting the microbiological results of the current study, the potential for recolonization of the pockets after this interval should be considered.

A previous review and meta-analysis indicated that LLLT showed only short-term additional benefits as an adjunct to SRP in reducing probing depth. The included studies described various differences in the output power and exposure time of applied LLLT in their protocols. The resulting energy dosage therefore ranged from 0.12 to 12 J per tooth in multiple sessions of irradiation, with an accumulative dosage of 1–30 J per site [40]. Ren et al. examined clinical parameters and collected supragingival plaque samples from periodontally compromised patients undergoing orthodontic treatment before and after adjunctive laser therapy [41]. Compared with the control group, the laser group displayed significantly milder gingival inflammation and fewer supragingival plaques at 1 month and 3 months. A recent study on LLLT focused on type 2 diabetes patients suffering from periodontitis. The percentage of moderate periodontal pockets was lower in the laser group than in the control group at six months [42]. Another trial confirmed the beneficial effect of this treatment modality on patients with periodontal compromised type 2 diabetes. Compared with those in the control area, the clinical parameters, PPD after one, three and six months and CAL after six months, were improved after adjunctive LLLT [43]. In the present investigation, both treatment groups presented considerable improvements in all the measures. According to earlier research, the clinical parameters were reassessed following a three-month healing period [28, 40]. The current findings, however, did not confirm a beneficial effect on clinical parameters with the additional use of lasers and the employed settings.

Standardized treatment guidelines for adjuvant low-level laser therapy in periodontal treatment have not yet been established. The results of this investigation do not support an adjuvant LLLT application in periodontal therapy owing to a lack of noteworthy clinical benefit following its application after scaling and root planning. To further explore this treatment modality, additional long-term studies with modified laser device parameters are advisable.

## Conclusion

Within the limitations of this study, adjuvant LLLT in periodontal treatment did not result in an additional improvement in the clinical parameters compared with those of the control group. Both treatment groups showed significant improvements in all investigated parameters after initial periodontal therapy. Microbiological analysis revealed a slightly greater reduction in *P. gingivalis* and *T. denticola* following LLLT, but the difference was not statistically significant. To date, there has been no unified protocol for the clinical application or treatment duration of LLLT in periodontal treatment. Future long-term clinical trials with different parameters are recommended to observe the effect of phototherapy in initial and supportive periodontal therapy.

## Data Availability

Data supporting the findings of this study are available within the manuscript.
